# Mutations in *Hedgehog Acyltransferase* (Hhat) Perturb Hedgehog Signaling, Resulting in Severe Acrania-Holoprosencephaly-Agnathia Craniofacial Defects

**DOI:** 10.1371/journal.pgen.1002927

**Published:** 2012-10-04

**Authors:** Jennifer F. Dennis, Hiroshi Kurosaka, Angelo Iulianella, Jennifer Pace, Nancy Thomas, Sharon Beckham, Trevor Williams, Paul A. Trainor

**Affiliations:** 1 Stowers Institute for Medical Research, Kansas City, Missouri, United States of America; 2 Department of Anatomy and Cell Biology, University of Kansas Medical Center, Kansas City, Kansas, United States of America; 3 Department of Anatomy and Neurobiology, Faculty of Medicine, Dalhousie University, Halifax, Nova Scotia, Canada; 4 Department of Cell Biology, Stem Cells, and Development, University of Colorado Denver, Aurora, Colorado, United States of America; Harvard Medical School, United States of America

## Abstract

Holoprosencephaly (HPE) is a failure of the forebrain to bifurcate and is the most common structural malformation of the embryonic brain. Mutations in *SHH* underlie most familial (17%) cases of HPE; and, consistent with this, *Shh* is expressed in midline embryonic cells and tissues and their derivatives that are affected in HPE. It has long been recognized that a graded series of facial anomalies occurs within the clinical spectrum of HPE, as HPE is often found in patients together with other malformations such as acrania, anencephaly, and agnathia. However, it is not known if these phenotypes arise through a common etiology and pathogenesis. Here we demonstrate for the first time using mouse models that *Hedgehog acyltransferase* (*Hhat*) loss-of-function leads to holoprosencephaly together with acrania and agnathia, which mimics the severe condition observed in humans. *Hhat* is required for post-translational palmitoylation of Hedgehog (Hh) proteins; and, in the absence of *Hhat*, Hh secretion from producing cells is diminished. We show through downregulation of the Hh receptor Ptch1 that loss of *Hhat* perturbs long-range Hh signaling, which in turn disrupts Fgf, Bmp and Erk signaling. Collectively, this leads to abnormal patterning and extensive apoptosis within the craniofacial primordial, together with defects in cartilage and bone differentiation. Therefore our work shows that *Hhat* loss-of-function underscrores HPE; but more importantly it provides a mechanism for the co-occurrence of acrania, holoprosencephaly, and agnathia. Future genetic studies should include *HHAT* as a potential candidate in the etiology and pathogenesis of HPE and its associated disorders.

## Authors Summary

Craniofacial anomalies account for approximately one third of all birth defects, and holoprosencephaly (HPE) is the most common structural malformation of the embryonic brain. HPE is a failure of the forebrain to bifurcate and is a heterogeneous disorder that is often found in patients together with other craniofacial malformations. Currently, it is not known if these phenotypes arise through a common etiology and pathogenesis, as the genetic lesions responsible for HPE have only been identified in about 20% of affected individuals. Here we demonstrate for the first time that *Hedgehog acyltransferase* (*Hhat*) loss-of-function leads to holoprosencephaly together with acrania and agnathia, which highlights the importance of Hh signaling in complex craniofacial disorders.

## Introduction

Holoprosencephaly (HPE) is a congenital malformation resulting from the failure of the forebrain to divide into left and right hemispheres [1], [2]. HPE occurs at a frequency of 1 in 10,000–20,000 live births, although affected embryonic individuals are thought to be as high as 1 in 250 pregnancies, making it the most common brain anomaly in humans [3], [4]. Within the clinical spectrum of HPE, a broad range of brain malformations are observed. Alobar holoprosencephaly is the most severe form, and in this case hemisphere bifurcation completely fails to occur resulting in the forebrain developing as a single holosphere together with a single cyclopic eye [5]. In milder instances such as lobar holoprosencephaly, near complete hemisphere separation occurs but cortical structures are hypoplastic and specific brain nuclei remain congruous [5]. It has long been recognized that the face predicts the brain [6] and HPE manifests with a range of craniofacial anomalies in humans. In extreme cases of HPE, cyclopia and a nasal proboscis can occur [3], [7]. In very mild forms, such as cebocephaly, only a single nostril or incisor might be present and the philtral ridges may be absent [8]. Thus phenotypically, HPE is a heterogeneous disorder and this is also true etiologically.

Exposure to environmental teratogens such as alcohol [9], [10] and retinoids [9] can result in HPE phenotypes. Gestational diabetes is also a factor as 1–2% of newborn infants of diabetic mothers exhibit HPE [11]. Genetically, HPE is similarly heterogeneous and is currently associated with mutations in at least 12 different loci encompassing multiple signaling pathways such as BMP, NODAL, ZIC, SIX, and SHH [4]. What is common amongst many of the loci and signaling pathways is that they play important roles in the development of the ventral brain and midline structures of the embryo. This is particularly true for Sonic Hedgehog (SHH) signaling.

Shh is a member of the Hedgehog (Hh) family of signaling molecules that also includes Indian (Ihh) and Desert Hedgehog (Dhh). Each Hh is a secreted glycoprotein that undergoes autoproteolytic cleavage and dual lipid post-translational modification to generate its proper active form. Autoproteolytic cleavage of Hh precursor molecules generates an N-terminal fragment (Hh-N) referred to as the mature form. Hh-N is then modified via the addition of a cholesterol moiety to its C-terminus [12], [13], followed by addition of a palmitoyl moiety to its N-terminus [14]. These lipid modifications are required for Hh protein multimerisation, distribution and activity.

To date, mutational analyses of human HPE, together with naturally occurring and engineered mouse mutants, have identified the genetic lesions responsible for only about 20% of individuals with HPE. Hence it is critical to identify additional candidate genes for the majority of patients whose genetic lesions remain unknown. Furthermore, HPE is a heterogeneous disorder and often found in patients together with other malformations such as acrania, anencephaly and agnathia and it is not known if these phenotypes arise through a common etiology and pathogenesis. Here we describe the *AP2-Cre* (“*Creface*”) mouse [15] as an insertional mutation in *Hhat*, and define *Hhat* as a novel HPE associated gene which can mechanistically explain the co-occurrence of HPE together with acrania and agnathia.

## Results

### “*Creface*” mice exhibit acrania-holoprosencephaly-agnathia

**Figure 1:**
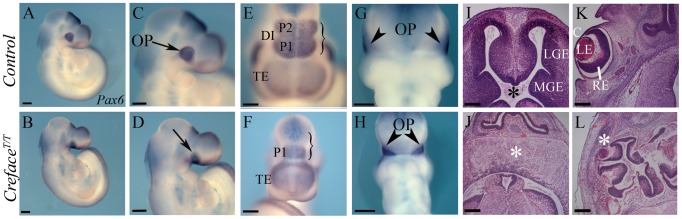
*Creface^T/T^* embryos exhibit brain anomalies. *Pax6* whole embryo *in situ* hybridization in E9.5 control (A, C, E, G) and *Creface^T/T^* (B, D, F, H) embryos demonstrating ventrally displaced optic cup (arrows, arrowheads) and agenesis of prosomere 2 in the diencephalon (parentheses) of mutant embryos. Hematoxylin and eosin stained transverse sections through the forebrain and eye regions of control (I, K) and *Creface^T/T^* (J, L) embryos reveal absence of the ventricle due to bifurcation failure (asterisk in I, J) and agenesis of the cornea in association with microphthalmia (asterisk in L) in mutants. Abbreviations: C, cornea; DI, diencephalon; LE, lens; LGE, lateral ganglionic eminence; MGE, medial ganglionic eminence; OP, optic placode; P, prosomere; RE, retina; TE, telencephalon. Scale bars: A–H 500 uM; I–L 200 uM.

“*Creface*” (*AP2-Cre*) is a transgenic line of mice [15] in which nuclear localizing *Cre* recombinase is driven by a specific *TFAP2A* enhancer element [16]. We discovered that interbreeding heterozygous *Creface^+/T^* mice failed to generate any post-natal viable homozygous *Creface^T/T^* animals. Therefore we investigated the etiology and pathogenesis of the *Creface^T/T^* mutant phenotype during embryogenesis. Morphological abnormalities in *Creface^T/T^* embryos are readily identifiable as early as E9.5. In contrast to control littermates, *Creface^T/T^* embryos exhibited smaller telencephalic hemispheres together with diencephalic and mesencephalic hypoplasia (Figure 1A, 1B). *Pax6* expression demarcates the telencephalon and prosomere (P) territories 1 and 2 of the diencephalon and *in situ* hybridization analyses with *Pax6* revealed the specific absence of P2 as well as abnormal neural morphology in E9.5 *Creface^T/T^* embryos (Figure 1A–1F). *Pax6* also labels the optic placode and interestingly, although present, the optic vesicles are displaced ventrally and medially in *Creface^T/T^* embryos (Figure 1C, 1D, 1G, 1H). At later stages of gestation, the forebrain in *Creface^T/T^* embryos often lacked a ventricular canal and instead persisted as a single–lobed or incompletely bifurcated neuroepithelium. In contrast, control littermates, displayed bifurcated hemispheres surrounding the forebrain ventricle (Figure 1I, 1J). Ocular anomalies in *Creface^T/T^* embryos manifested as microphthalmia but in addition, the eye often remained embedded in grossly disorganized brain tissue and the lack of contact with the surface ectoderm resulted in a failure to form tissues such as the cornea (Figure 1K, 1L).

**Figure 2:**
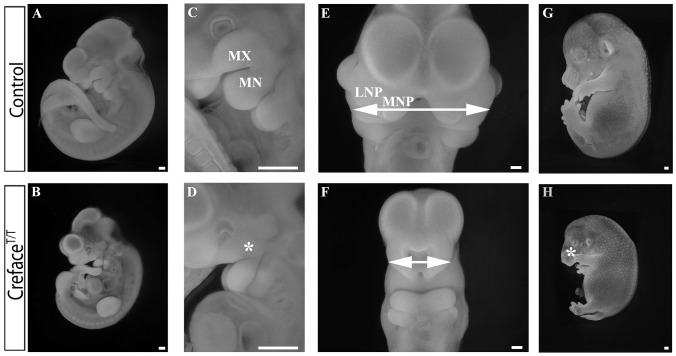
*Creface^T/T^* embryos exhibit general facial prominence hypoplasia. Fluorescent images of whole DAPI stained E10.5 control (A, C, E) and *Creface^T/T^* (B, D, F) embryos highlighting the pharyngeal (asterisk) and midfacial hypoplasia evident in the form of a single nasal slit (double arrows) in mutant embryos. Bright field images of E14.5 control (G) and *Creface^T/T^* (H) embryos illustrating the severe frontonasal defects in mutant embryos (asterisk). Abbreviations: LNP, lateral nasal prominence; MN, mandibular prominence; MNP, medial nasal prominence; MX, maxillary prominence. Scale bars: 200 uM.

E10.5 *Creface^T/T^* mutant embryos are noticeably smaller in size than control littermates and exhibit more prominent craniofacial abnormalities (Figure 2A, 2B). In particular, the frontonasal region of the embryo as defined by the medial nasal prominences and spacing between the bilateral nasal slits is dramatically reduced in size to the extent that only a single slit is present in *Creface^T/T^* mutant embryos (Figure 2C, 2D). Craniofacial anomalies in *Creface^T/T^* embryos are not limited to the brain and frontonasal region as the maxillary and mandibular components of the first pharyngeal arch are also hypoplastic at E9.5–10.5 (Figure 2E, 2F). This manifests in E14.5 mutant embryos as a narrow protruding midface, together with more severely pronounced maxillary and mandibular hypoplasia (Figure 2G, 2H). In addition to craniofacial defects, mutant embryos exhibited limb defects including oligodactyly (Figure 2G, 2H and data not shown). E14.5 *Creface^T/T^* embryos displayed considerable edema with the outer layer of skin being displaced from the body cavity, most likely due to defects in lymphatic development (Figure 2G, 2H). Large areas of blood pooling were also often observed in the anterior region of the embryos, which may be indicative of more general vascular anomalies. These lymphatic and vascular anomalies progressively worsened coinciding with embryonic lethality prior to birth.

**Figure 3:**
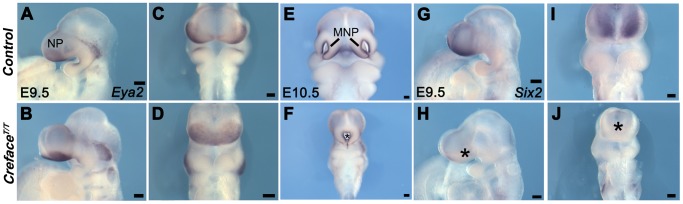
*Creface^T/T^* embryos exhibit frontonasal placode patterning defects. *Eya2* (A–F) and *Six2* (G–J) whole embryo *in situ* hybridization in E9.5 and E10.5 control (A, C, E, G, I) and *Creface^T/T^* (B, D, F, H, J) embryos showing agenesis of frontonasal mesenchyme (I, J), nasal placode fusion (C, D) and formation of a single nasal slit in mutant embryos (asterisks). Abbreviations: NP, nasal placode. Scale bars: A and G 500 uM; all others 200 uM.

**Figure 4:**
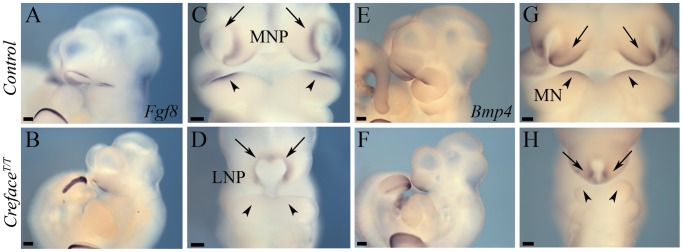
*Creface^T/T^* embryos exhibit frontonasal and pharyngeal epithelial patterning defects. *Fgf8* (A–D) and *Bmp4* (E–H) whole embryo *in situ* hybridization in E10.5 control (A, C, E, G) and *Creface^T/T^* (B, D, F, H) embryos highlighting the midfacial hypoplasia, single nasal slit (arrows) and abnormal proximodistal pharyngeal arch patterning (arrowheads) defects in mutant embryos. Scale bars: 200 uM.

In the current analysis we concentrated on the molecular and structural changes associated with the *Creface^T/T^* craniofacial defects. In control embryos *Eya2* bilaterally demarcates the nasal placode ectoderm (Figure 3A, 3C, 3E) while *Six2* is expressed bilaterally in the mesenchyme of each medial nasal prominence (Figure 3G, 3I). In E9.5–10.5 *Creface^T/T^* mutants, there is a single continuous central domain of placodal *Eya2* activity (Figure 3B, 3D, 3F), while *Six2* expression is absent from midline tissues (Figure 3H, 3J). This is consistent with frontonasal agenesis, nasal placode fusion and a single nasal pit/slit in *Creface^T/T^* mutant embryos (Figure 2C, 2D). We next examined the signaling molecules Fgf8 and Bmp4, which are known to regulate craniofacial development [17]. At E10.5, *Fgf8* normally labels the epithelium flanking the nasal pits almost uniformly (Figure 4A, 4C), while *Bmp4* marks only specific ventral domains of the nasal prominences (Figure 4E, 4G). However, *Creface^T/T^* mutants display single continuous domains of epithelial *Fgf8 and Bmp4* expression (Figure 4B, 4D, 4F, 4H). These findings are also consistent with frontonasal agenesis, nasal placode fusion and a single nasal pit/slit in *Creface^T/T^* mutant embryos.

**Figure 5:**
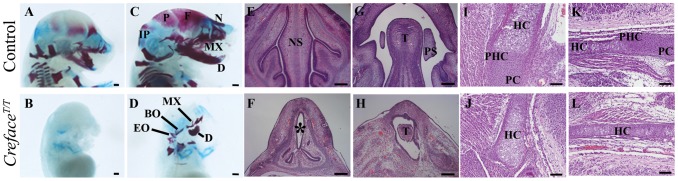
*Creface^T/T^* embryos exhibit major cranioskeletal malformations. Alcian blue carilage and Alizarin red bone staining of E15.5 (A, B) and E17.5 (C, D) control (A, C) and *Creface^T/T^* (B, D) embryos highlighting acrania, agnathia and general cranioskeletal agenesis in mutant embryos. Hematoxylin and eosin stained sections through the nasal septum region (E, F), palatal mesenchyme (G, H) exoccipital (I, J) and basioccipital (K, L) bones of E14.5 control (E, G) and *Creface^T/T^* (F, H) and E17.5 control (I, K) and *Creface^T/T^* (J, L) embryos illustrating tissue agenesis (asterisk) and hypoplasia as well as defects in endochondral ossification in mutant embryos. Abbreviations: BO, basioccipital; D, dentary; EO, exoccipital; F, frontal; HC, hypertrophic chondrocytes; IP, interparietal; MX, maxilla; N, nasal; NS, nasal septum; P, parietal, PC, proliferating chondrocytes; PHC, prehypotrophic chondrocytes; PS, palatal shelves; T, tongue. Scale bars: A–D 500 uM; I–L 100 uM; E–H 200 uM.

To characterize any defects in skeletogenesis in *Creface^T/T^* mutants, we stained embryos at E15.5 and 17.5 (Figure 5) with Alcian blue and Alizarin red to label cartilage and bone, respectively. At E15.5 control embryos exhibited considerable domains of differentiated cartilage within the head, particularly within the developing cranium, ear and nasal regions as evidenced by extensive blue tissue staining (Figure 5A). Ossification was clearly evident in multiple elements including the frontal and parietal bones but was particularly well advanced in components of the viscerocranium including the premaxillary, maxillary and dentary bones. In marked contrast, E15.5 *Creface^T/T^* littermates demonstrated a complete absence of Alizarin red stained ossified bone within the skull and jaw (Figure 5B). Mutant embryos also displayed considerably diminished chondrogenesis of calvarial, nasal and otic mesenchyme (Figure 5B). Not only were cranial elements such as the nasal cartilage hypoplastic or missing, but staining of cartilage in the vertebral column was also absent. Section histology revealed that compared to control littermates, which have bifurcated continuous nasal cavities separated by a nasal septum, E14.5 *Creface^T/T^* embryos lacked a nasal septum and possessed only a single yet discontinuous nasal cavity (Figure 5E, 5F). In addition, although the palatal shelves were readily identifiable adjacent to the tongue in control embryos, this was not the case in *Creface^T/T^* littermates, and is indicative of a defect in vertical extension and medial growth of the palatal shelves toward the midline (Figure 5G, 5H). The oral cavity was considerably narrower in mutant embryos and the tongue was hypoplastic.

By E17.5, cranioskeletal mineralization was almost complete in control embryos. The frontal, parietal, interparietal, supraoccipital, exoccipital and temporal bones that contribute to the neurocranium are nearly fully ossified as were the nasal, premaxilla, maxilla, jugal and dentary bones within the viscerocranium (Figure 5C). In contrast *Creface^T/T^* littermate embryos exhibited acrania as the frontal parietal, interparietal and supraoccipital, bones were all completely absent (Figure 5D). The nasal cartilage that was present was a tube-shaped structure that was devoid of ossification. The basioccipital and exoccipital bones that constitute the base of the skull were present, but were dramatically reduced in size and abnormally shaped. E17.5 *Creface^T/T^* embryos also display agnathia as we observed the presence of only rudimentary premaxilla, maxilla and dentary bones. The dentary bones lacked all processes including the condyle, angular and coronoid processes. Furthermore *Creface^T/T^* embryos also exhibited disrupted tooth development as incisor and alveolar molar tooth primordia which were readily identifiable in control embryos, were absent from mutant littermates (data not shown).

Given the extensive nature of cranioskeletal anomalies in *Creface^T/T^* embryos, we characterised their specific pathogenesis in more detail. During endochondral ossification proliferating chondrocytes at distal ends of a skeletal element undergo hypertrophy and dramatically increase in size. As development proceeds, hypertrophic chondrocytes undergo apoptosis and are replaced by invading osteoblasts (reviewed in [18]). Sagittal sections through the exoccipital and basioccipital bones of E15.5 *Creface^+/T^* embryos revealed the presence of proliferating chondrocytes at the distal end of the element (Figure 5I, 5K). These cells were located adjacent to a domain of prehypertrophic chondrocytes which were in turn were flanked by noticeably larger hypertrophic chondrocytes positioned centrally within each element. In contrast, sections of *Creface^T/T^* mutants revealed a significant perturbation in chondrocyte development. The skeletal elements were reduced in size and this appeared to be associated with a diminished zone of proliferating chondrocytes (Figure 5J, 5L).

### *Hedgehog acyltransferase (Hhat)* is disrupted in *Creface^T/T^* mutants

The constellation of craniofacial abnomalities observed in *Creface^T/T^* embryos is consistent with acrania-holoprosencephaly-agnathia. Holoprosencephaly and limb anomalies including digit oligodactyly are classic features of perturbed Hh signaling during early embryogenesis. We therefore hypothesized that the *AP2-Cre* transgene used to generate *Creface* mice, inserted into or interfered with a locus important for Hh signaling. However, to rule out the possibility that the insertion occurred in Hh signaling genes previously associated with HPE, we initially performed complementation tests by intercrossing *Creface^+/T^* mice with *Shh^+/−^* [19] and *Ptch1^+/−^* [20] mice. None of the *Creface^+/T^;Shh^+/−^*, or *Creface^+/T^;Ptch1-LacZ^+^* embryos harvested at E17.5–18.5 displayed craniofacial anomalies consistent with HPE, acrania or agnathia (data not shown). This indicated that the *Creface^T/T^* phenotype was not the result of transgene integration into *Shh*, or *Ptch1* genomic loci.

Given that the genetic lesions responsible for HPE have only been identified in about 20% of affected individuals, there is considerable interest in discovering new genes that contribute to its etiology and pathogenesis. This is particularly true where HPE is a subcomponent of more complex syndromes. Therefore, using a PCR-based method for DNA walking and mapping known as Vectorette, we determined that the *Creface* transgene had integrated into intron 9 of *Hedgehog acyltransferase* (*Hhat*) at nucleotide position 22949 (Figure S1A). To further verify that *Hhat* was indeed the gene disrupted, we genotyped E10.5 embryos obtained from intercrosses of *Creface^+/T^* mice using control and transgene-specific reactions. Genotyping of the endogenous *Hhat* intron 9 region produced a 1.4 kb DNA band from both wild-type and heterozygous (*Creface^+/T^*) embryos, but did not produce a band from mutant (*Creface^T/T^*) embryos as expected (Figure S1B). Furthermore, using a primer designed to recognize the *Creface:Hhat* fusion, this enabled amplification of an integration specific 250 bp fragment from *Creface^+/T^* heterozygous and *Creface^T/T^* mutant embryos but not from wild-type embryos (Figure S1B). Collectively, these results confirmed that the *Creface* transgene integrated within intron 9 of *Hhat*. It is well recognized that transgenes integrate into host genomes as concatamers and can disrupt endogenous gene expression [21]–[23]. Consistent with this we were unable to RT-PCR amplify full length *Hhat* using RNA obtained from *Creface^T/T^* mutant embryos, in contrast to wild-type and *Creface^+/T^* heterozygous embryos (Figure S1C).

**Figure 6:**
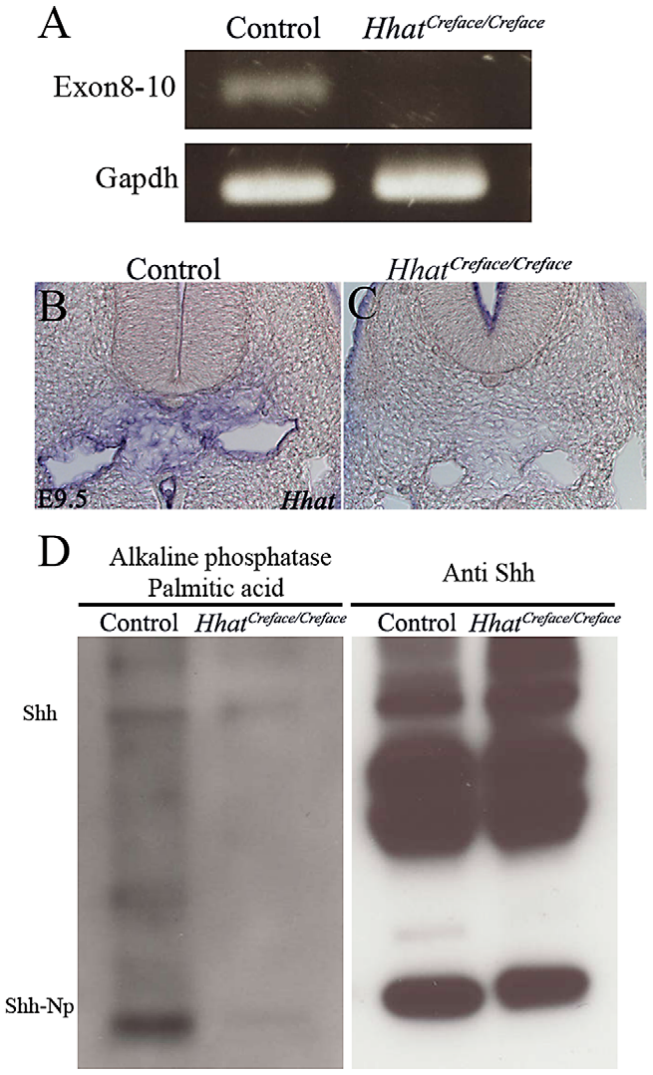
*Hhat* loss-of-function perturbs gene expression and palmitoylation of *Shh.* * Hhat* gene expression analysis by RT-PCR (A) and *in situ* hybridization (B, C) showing diminished or absent activity in *Hhat^Creface/Creface^* mutant embryos compared to controls, particularly with respect to expression in the mesenchymal cells surrounding the notochord. (D) Palmitoylation assay performed using MEFs from control and *Hhat^Creface/Creface^* mice. *Hhat^Creface/Creface^* MEFs exhibited a considerable reduction in palmitoylated *Shh* both in unprocessed (Shh) and processed (Shh-Np) protein forms (left panel) despite western blotting highlighting equivalent amounts of *Shh* protein in mutant compared to control mice (right panel). The expression of *Hhat* mRNA according to the mutation site was absent in *Hhat^Creface/Creface^* mutant (B). *In situ* hybridization revealed that *Hhat* mRNA level of *Hhat^Creface/Creface^* mice (C, right panel) are impaired at the mesenchyme cells around the notochord compared with control mice (C, left panel).

To examine the expression of *Hhat* in control and mutant embryos, we generated a riboprobe corresponding specifically to exons 8–10 of *Hhat* (Figure 6A). Through *in situ* hybdrization of E9.5–10.5 embryos followed by sectioning, we observed that *Hhat* is expressed in and around the notochord ventral to the neural tube in wild-type embryos. In contrast, the level of *Hhat* expression was considerably diminished or absent in *Creface^T/T^* mutant littermate embryos (Figure 6B, 6C). This indicates that *Hhat* activity is disrupted through *Creface* transgene insertion. These data clearly validate that integration of the *Creface* transgene physically disrupts *Hhat* rendering the gene non-functional, which results in the morphological defects characteristic of *Creface^T/T^* mutant mice. *Creface^+/T^* heterozygous and *Creface^T/T^* homozygous mice will herein be referred to as *Hhat^+/Creface^* and *Hhat^Creface/Creface^* respectively.

### Loss of Hhat function disrupts Shh signaling

*Hhat* encodes an acyltransferase that palmitoylates Hh proteins [14]. This fatty acid modification increases the potency of Hh signaling *in vitro* [24] and is also required for long-range Hh signaling *in vivo* [25]. Consistent with this, *Drosophila* mutants lacking Hh palmitoylation exhibit patterning defects in Hh-responsive cells [26]–[29]. We therefore hypothesized that *Hhat* loss-of-function should disrupt the palmitoylation of Shh and perturb its signaling effects in *Hhat^Creface/Creface^* embryos. To test this, we generated mouse embryonic fibroblast (MEFs) from control and *Hhat^Creface/Creface^* embryos, transfected each with a *Shh* expression vector, and incubated them with palmitic acid for 12 hours. Incorporated palmitic acid was then tagged with biotin. Protein extraction, followed by western blotting with an anti Shh antibody and alkaline phosphatase streptavidin staining, confirmed that despite the presence of similar levels of total Shh protein, the degree of palmitoylation in *Hhat^Creface/Creface^* MEFs was considerably diminished compared to controls (Figure 6D). This clearly demonstrates that the functionality of Hhat is compromised in *Hhat^Creface/Creface^* embryos.

**Figure 7:**
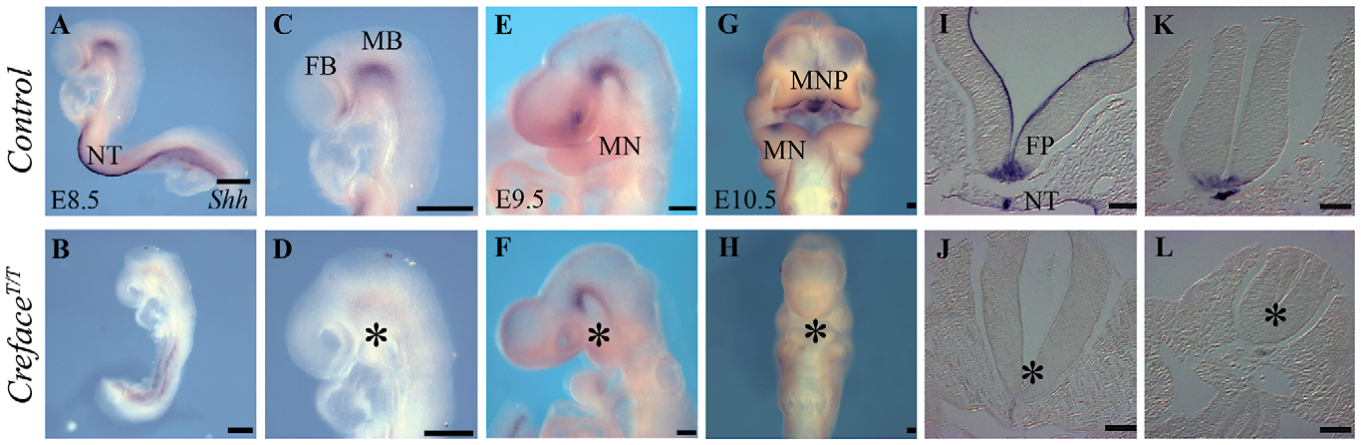
*Shh* expression is altered in *Hhat^Creface/Creface^* embryos. *Shh in situ* hybridization in whole E8.5–10.5 control (A, C, E, G) and *Hhat^Creface/Creface^* (B, D, F, H) embryos illustrating the downregulation or loss of *Shh* expression in mutant embryos. Sections through the neural tube and notochord of E9.5 control (I, K) and *Hhat^Creface/Creface^* (J, L) highlighting the absence of *Shh* expression in the floor plate of mutant embryos (asterisk). Abbreviations: FB, forebrain; FP, floor plate; MB, midbrain; NT, notochord. Scale bars: A–D 200 uM; E 500 uM; F–H 200 uM; I–L 50 uM.

Our expectation from *Hhat* loss-of-function in concert with the holoprosencephaly phenotype was that the lack of palmitoylation should perturb the spatiotemporal activity of Shh signaling throughout *Hhat^Creface/Creface^* embryos. Hence we initially examined the expression of *Shh* via *in situ* hybridization in control and mutant embryos. Compared to control littermates, *Shh* was expressed in the notochord in E8.5 and E9.5 *Hhat^Creface/Creface^* embryos albeit at slightly reduced levels but was absent from the ventral telencephalon and floor plate (Figure 7A–7F, 7I–7L). *Shh* expression was also absent from the branchial arch (BA) ectoderm and pharyngeal endoderm of E10.5–11.5 *Hhat^Creface/Creface^* embryos (Figure 7G, 7H). Shh activity in tissues such as the ventral forebrain and branchial arch ectoderm is induced by and dependent upon prior expression of *Shh* in the notochord and endoderm respectively [30], [31]. Thus the alterations to *Shh* expression in *Hhat^Creface/Creface^* embryos were indicative of specific disruptions to Shh signaling gradients as a consequence of *Hhat* loss-of-function and lack of Shh palmitoylation.

**Figure 8:**
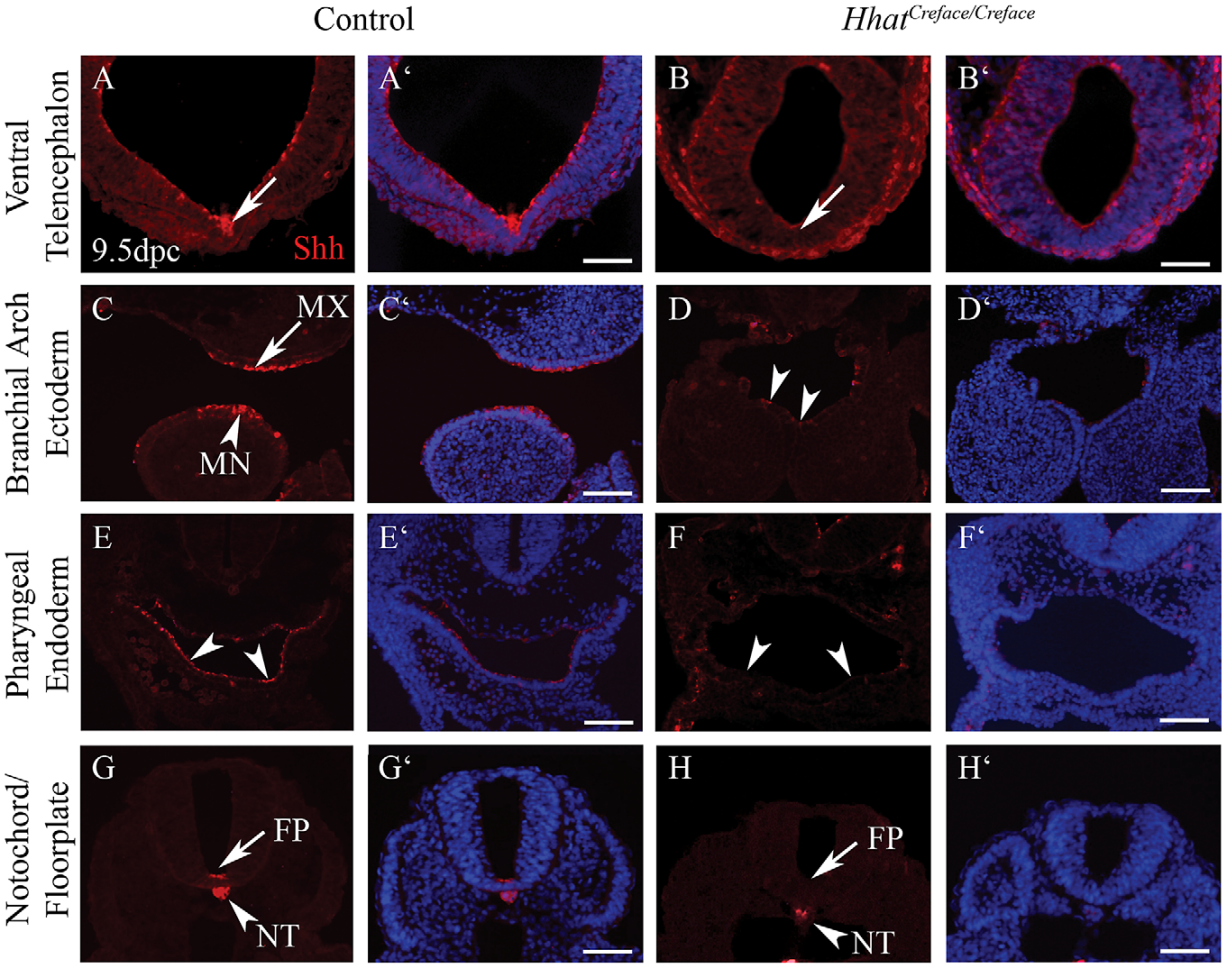
Shh activity is disrupted in *Hhat^Creface/Creface^* embryos. Shh immunostaining (red) and DAPI nuclei staining (blue) of transverse sections of E10.5 control and *Hhat^Creface/Creface^* embryos demonstrating the absence or reduction of Shh activity in the ventral telencephalon (A–B′; arrows), branchial arch ectoderm (C, C′ arrows; D, D′ arrowheads), pharyngeal endoderm (E–F′, arrowheads) and floor plate (G–H′, arrows) of mutant embryos. Scale bars: 200 uM.

To confirm this, we characterized the distribution of Shh protein in E9.5 embryos (Figure 8). Control embryos exhibited Shh activity in the ventral telencephalon, branchial arch ectoderm, pharyngeal endoderm, notochord and floor plate (Figure 8A, 8C, 8E, 8G). However, in *Hhat^Creface/Creface^* embryos, Shh protein was absent from all these sites with the exception of the notochord (Figure 8B, 8D, 8F, 8H). These findings are consistent with our observations of *Shh* expression via *in situ* hybridization (Figure 7) and correlate with the localization of *Hhat* activity. Thus our results indicate that localized tissue specific production of Shh can occur in *Hhat^Creface/Creface^* embryos, however the failure to induce secondary domains of activity suggests a disruption in long-range Shh signaling gradients as a function of perturbed palmitoylation.

### *Hhat^Creface/Creface^* embryos have decreased Patched1 activity

**Figure 9:**
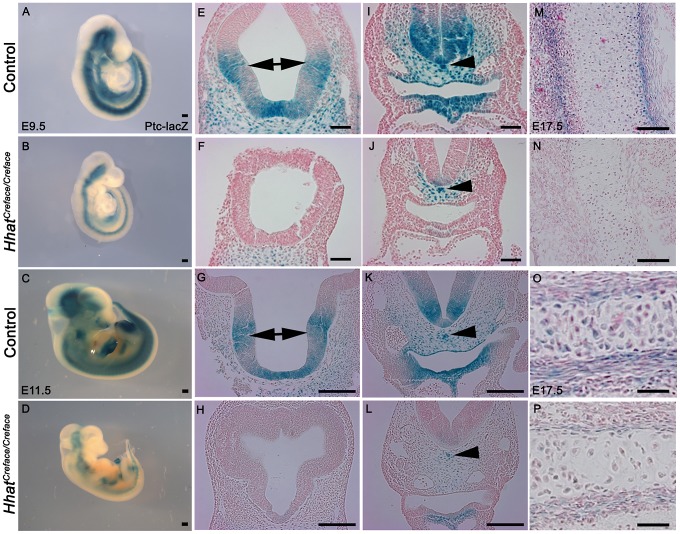
*Patched (Ptch1)* activation is diminished in *Hhat^Creface/Creface^* embryos. Ptch1-LacZ reporter staining in whole and transverse sectioned E9.5 and 11.5 control (A, C, E, G, I, K) and *Hhat^Creface/Creface^* (B, D, F, H, J, L) embryos revealed a reduction in Shh signaling gradients and range throughout the telencephalon (E–H; double arrows), spinal cord, peri-notochord mesenchyme (arrowhead) and pharyngeal tissues (I–L) in mutant embryos. Ptch1-LacZ reporter staining of cranial sagittal sections through the exoccipital and basioccipital bones from E17.5 control (M, O) and *Hhat^Creface/Creface^* (N, P) embryos illustrate the reduction of Shh signaling during endochondral ossification of cranioskeletal elements. Scale bars: A and B 200 uM; C and D 500 uM; E,F,I,J 50 uM; G,H,K,L 100 uM; M,N 100 uM; O,P 200 um.

Ptch1 is a receptor for Hh ligands and its activity is widely used as a read out of the gradient and range of Hh signaling. Therefore to better document the degree of spatial perturbation of Hh signaling in *Hhat^Creface/Creface^* embryos, we outcrossed *Hhat^+/Creface^* mice to *Ptch1-LacZ* mice [20] and used the LacZ reporter as a measure of Ptch1 activity. E9.5–11.5 *Hhat^+/Creface^;Ptch1-LacZ^+^* embryos exhibit intense LacZ expression in the ventral telencephalon (arrows), pharyngeal endoderm, notochord (arrowheads) and surrounding mesenchyme as well as in the floorplate (Figure 9A, 9C, 9E, 9G, 9I, 9K). Often, a gradient of Ptch1 activity is present within or adjacent to these sites of Shh synthesis, which reflects the extensive range of Hh signaling in each of these regions (Figure 9I, 9K). In contrast, Ptch1-LacZ activity is considerably reduced in *Hhat^Creface/Creface^* embryos (Figure 9B, 9D). More specifically these mutant embryos lack LacZ expression in the ventral telencephalon, branchial arch ectoderm, pharyngeal endoderm and floor plate (Figure 9F, 9H, 9J, 9L). LacZ activity in the mesenchyme surrounding the notochord is present in *Hhat^Creface/Creface^* mutants, but is considerably reduced compared to control littermates (Figure 9I–9L). These results specifically highlight the spatiotemporal diminishment of the gradient and range of Shh activity in *Hhat^Creface/Creface^* embryos.

*Hhat* is required for the palmitoylation of all Hh proteins, not just Shh. Furthermore the cartilage and bones which are disrupted in *Hhat^Creface/Creface^* embryos are dependent upon the actions of both *Shh* [19] and *Indian hedgehog (Ihh)* [32]. Consistent with this *Shh^−/−^* mutant embryos exhibit agenesis of the calvarial bones, while loss of Ihh results in reduction of cranial bone size and all markers of osteodifferentiation during endochondral as well as membranous ossification [33]. Therefore we also documented the activity of Ptch1 during ossification to determine if global Hh signaling was impaired during cranioskeletal differentiation and if this also contributed to the pathogenesis of craniofacial anomalies in *Hhat^Creface/Creface^* embryos. In sections of E17.5–18.5 control embryos, strong Ptch1-LacZ activity was observed in the perichondrium and chondrocytes within cranioskeletal elements such as the exoccipital and basioccipital bones (Figure 9M, 9O). In contrast, Ptch1-LacZ activity was apparently absent from the exoccipital bone in *Hhat^Creface/Creface^;Ptch1-LacZ^+^* mutants (Figure 9N) and was considerably reduced in the basioccipital bone (Figure 9P). The disruptions in Ptch1 activity are indicative of a global diminishment of Hh signaling in *Hhat^Creface/Creface^* embryos. Our results therefore demonstrate a direct *in vivo* requirement for *Hhat* in establishing Hh signaling gradients during early and late stages of embryogenesis. Thus perturbation of Hh signaling directly underpins the etiology of cranioskeletal defects in *Hhat^Creface/Creface^* embryos.

### Neural crest lineages are altered in *Hhat^Creface/Creface^* mutants

*Hhat^Creface/Creface^* embryos display decreased Shh signaling in the ventral telencephalon and branchial arches from E8.5–10.5. Temporally, this coincides with cranial neural crest cell migration and colonisation of the frontonasal, maxillary and mandibular facial prominences. Neural crest cells generate most of the cartilage, bone and connective tissue in the head and face [34], [35] and *Shh* is known to be required for their proper migration and survival [36]–[39]. We hypothesized that defects in neural crest cell development could therefore be a major contributing factor to the pathogenesis of cranioskeletal anomalies in *Hhat^Creface/Creface^* embryos. Hence, we compared the formation, migration and survival of neural crest cells in control and *Hhat^Creface/Creface^* embryos. No obvious defects in the initial specification of cranial neural crest cells were observed from *in situ* hybridization analyses with general neural crest cell markers such as *Snail1* and *Crabp1* (Figure S2A–S2H), or more lineage specific markers such as *Sox9* and *Sox10* (Figure S2I–S2P). Additionally, we performed lineage tracing analyses using *Wnt1-Cre* together with *Rosa26R* mice to permanently label neural crest cells. In E9.5–10.5 control embryos, streams of LacZ positive neural crest cells migrated into and colonized the developing frontonasal prominences as well as the pharyngeal arches (Figure S3A, S3B). In *Hhat^Creface/Creface^;Wnt1-Cre^+^;R26R^+^* embryos LacZ positive neural crest cells were present in similar patterns to control littermates, further demonstrating that there was no significant defect in neural crest cell formation, migration or colonization of the facial prominences and pharyngeal arches in *Hhat^Creface/Creface^* embryos (Figure S3C, S3D). However, the patterns of LacZ staining highlighted the hypoplasia of medial, lateral, maxillary and mandibular facial prominences in *Hhat^Creface/Creface^* embryos, and also revealed gross anomalies in development of the cranial ganglia as the trigeminal appeared to be hypoplastic, and the hypoglossal and vagal ganglia were fused (Figure S3C, S3D).

### *Hhat^Creface/Creface^* embryos exhibit cranial ganglia defects

Cranial ganglia form part of the peripheral nervous system and are derived from both neural crest and sensory placode cells [40]. The gross cranial ganglia defects observed via lacZ staining in E10.5 *Hhat^Creface/Creface^;Wnt1-Cre^+^;R26R^+^* embryos were confirmed through anti-neurofilament (2H3) immunostaining (Figure S4). Not only were the cranial ganglia and in particular the trigeminal hypoplastic, but interestingly, immunostaining more discretely revealed that the maxillary branch of the trigeminal was consistently narrower in *Hhat^Creface/Creface^* embryos compared to control littermates (Figure S4A, S4D). This correlates with maxillary hypoplasia in mutant embryos (Figure 2A–2D). In addition, the hypoglossal and vagal ganglia are aberrantly fused and the oculomotor nerve which is readily identifiable in control embryos is missing in *Hhat^Creface/Creface^* littermates.

The dual origin of cranial ganglia from neural crest and ectodermal placode cells prompted us to examine whether defects in development of either progenitor population underpinned the ganglia defects in *Hhat^Creface/Creface^* mutants. E10.5 embryos were processed via *in situ* hybridization for *Sox10 and Eya2*, which demarcate neurogenic neural crest and sensory placode cells respectively. Although the spatiotemporal patterns of *Sox10* and *Eya2* expression were quite similar between control and mutant embryos, the domains of activity highlighted the hypoplasia of the trigeminal ganglia and fusion of the hypoglossal and vagal ganglia in *Hhat^Creface/Creface^* embryos (Figure S4B, S4C, S4E, S4F). Collectively, these results indicate that *Hhat* loss-of-function and disrupted Hh signaling does not globally affect the induction or migration of neural crest and sensory placode cells. However, hypoplasia and aberrant fusion of the cranial ganglia as evidenced by both neural crest (*Sox10*) and sensory placode (*Eya2*) markers suggested that Hh signaling plays a regionalized role in the development and patterning of both progenitor cell populations.

### *Hhat^Creface/Creface^* embryos exhibit increased apoptosis

**Figure 10:**
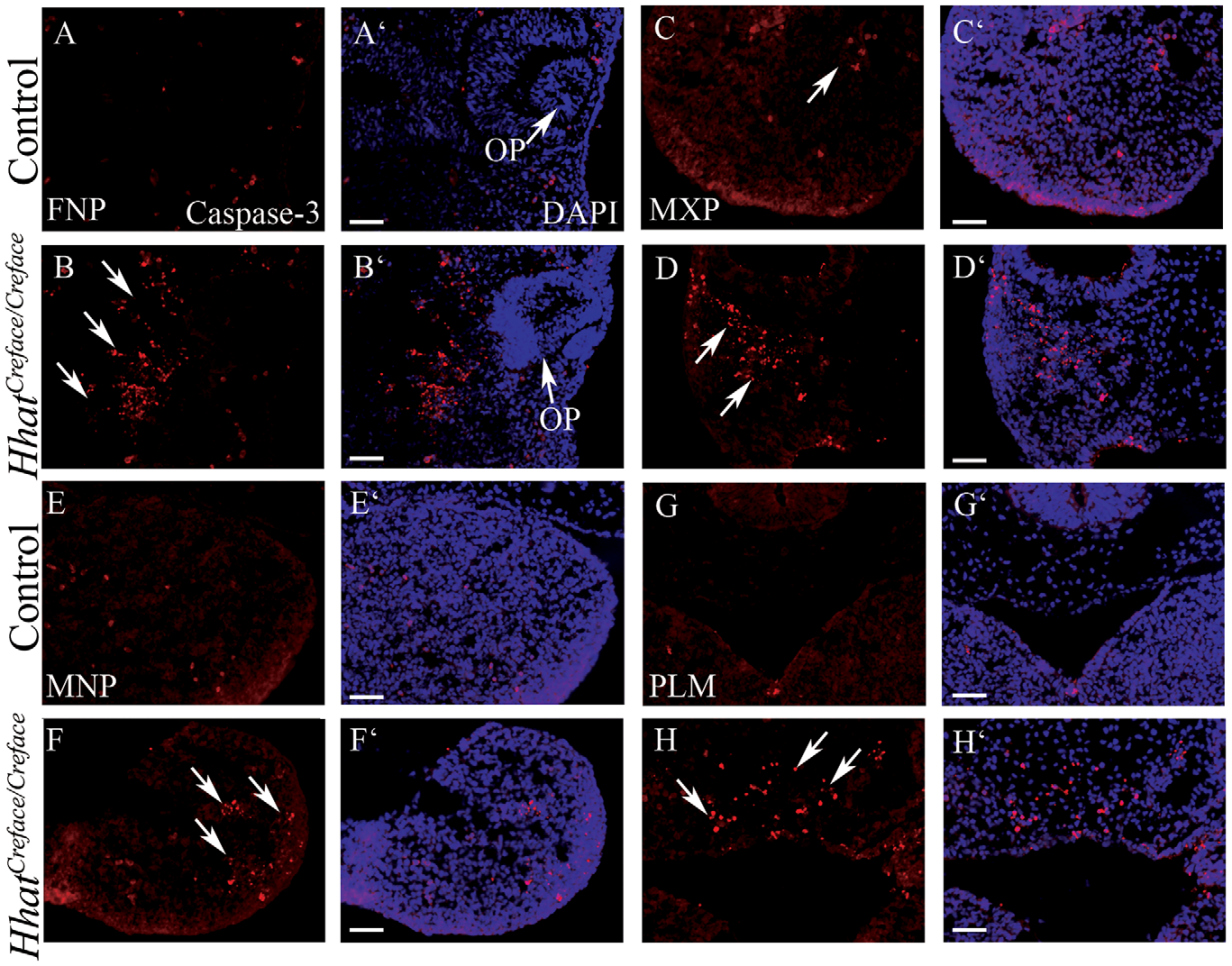
*Hhat^Creface/Creface^* embryos exhibit increased cell death in craniofacial mesenchyme. Caspase3 immunostaining (red) and DAPI (blue) nuclear staining of transverse sectioned E10.5 control (A, A′, C, C′, E, E′, G, G′) and *Hhat^Creface/Creface^* (B, B′, D, D′, F, F′, H, H′) embryos revealed elevated levels of apoptotic cells (arrows) in the frontonasal (A–B′), maxillary (C–D′) and mandibular (E–F′) prominences as well as in the palatal mesenchyme (G–H′) of mutant embryos. Abbreviations: FNP, frontonasal process; PLM, palatal mesenchyme. Scale bars: 200 uM.

Hypoplasia of the facial prominences together with the trigeminal ganglia, suggested that Hhat - through its effects on mediating the gradient and range of Hh signaling - may play a critical role in progenitor cell survival. Consistent with this idea, Shh has been shown to be critical for neural crest cell survival during craniofacial morphogenesis [36], [37], [41]. Therefore we examined whether there were any alterations in progenitor cell survival in *Hhat^Creface/Creface^* embryos by characterizing the spatiotemporal differences in cell death in control versus mutant embryos. Using the apoptotic marker cleaved-Caspase3, we observed a considerable increase in apoptotic cells in the neural crest cell derived frontonasal, maxillary, mandibular and prospective palatal mesenchyme of E9.5–10.5 *Hhat^Creface/Creface^* embryos compared to control littermates (Figure 10A–10H). In contrast, we did not observe any significant differences in the number of proliferative cells using the marker phospho-Histone 3 (data not shown). Our data demonstrates that *Hhat* loss-of-function perturbs Hh signaling during early embryogenesis resulting in increased apoptosis in neural crest cell derived craniofacial mesenchyme. This underscores hypoplasia of the facial prominences during early embryogenesis and together with defects in differentiation, subsequently contributes to the characteristic acrania-holoprosencephaly-agnathia malformations observed in *Hhat^Creface/Creface^* embryos.

### *Hhat^Creface/Creface^* embryos exhibit altered pharyngeal arch patterning

In addition to regulating cell survival, Hh signaling also plays important roles in governing branchial arch patterning. In fact, branchial arch ectoderm-derived Shh signaling, regulates downstream targets such as *Fgf8* and *Bmp4*, which respectively influence proximal versus distal pharyngeal arch and jaw patterning. *Fgf8* is normally expressed in the proximal maxillo-mandibular ectoderm of E9.5–10.5 embryos and is flanked (Figure 4A, 4C) distally within the mandible by an adjacent ectoderm domain of *Bmp4* (Figure 4E, 4G). Compared to control littermates, *Fgf8* (Figure 4B, 4D) and *Bmp4* (Figure 4F, 4H) expression were absent from the first pharyngeal arch ectoderm of E10.5 *Hhat^Creface/Creface^* embryos demonstrating their dependence on Hh signaling. Both Bmp and Fgf signaling are known to play critical temporal and tissue context dependent roles in cell proliferation and survival. Hence we examined the extent of their respective roles in the pathogenesis of craniofacial anomalies in *Hhat^Creface/Creface^* mutants during early embryogenesis.

Bmps transduce signals by binding to complexes of type I and II serine/threonine kinase receptors which then activate canonical signaling via receptor Smads (R-Smads) 1, 5 and 8. Immunostaining with phospho-SMAD1/5/8 revealed no obvious alterations in SMAD signaling in the frontonasal, maxillary or mandibular mesenchyme of E10.5 *Hhat^Creface/Creface^* embryos compared to control littermates (data not shown). The lack of any significant change in SMAD signaling is likely due to redundancy with other Bmp signals such as *Bmp7* and furthermore is consistent with previous analyses that conditionally deleted *Bmp4* from the branchial arch ectoderm [42].

In contrast, FGF signaling functions through membrane bound receptors which activate downstream effectors, such has mitogen-activated protein kinase (MAPK)/extracellular signal-regulated kinase (ERK). We observed a considerable decrease in both the domain and level of phosphorylated Erk1/2 staining within the frontonasal, maxillary and mandibular mesenchyme of *Hhat^Creface/Creface^* mutant embryos compared to control littermates (Figure S5A, S5B). This indicates that diminished Erk1/2 signaling impacts upon neural crest cell survival and contributes to hypoplasia of the facial prominences underpinning the pathogenesis of craniofacial anomalies in *Hhat^Creface/Creface^* embryos. Consistent with this, conditional deletion of *Erk1/2* in neural crest cells results in extensive agenesis of the neural crest cell derived craniofacial skeleton (P.A.T. unpublished). Furthermore, activation and repression of Shh signaling has been shown to respectively increase and decrease the levels of phosphorylated Erk1/2 *in vitro* [43]. Taken together with our *in vivo* data, this collectively highlights the vital role played by Hh-Fgf-Erk signaling in promoting neural crest cell survival and preventing apoptosis during normal craniofacial morphogenesis and in the pathogenesis of craniofacial anomalies in *Hhat^Creface/Creface^* embryos.

## Discussion

### *Hhat^Creface/Creface^*: a model of acrania-holoprosencephaly-agnathia

*SHH* was the first locus mapped in association with HPE in affected patients [1], [2] and knockouts of *Shh* in mice recapitulate the characteristics of HPE observed in human individuals. Mutations in Hh signaling pathway members, such as *Patched1 (Ptch1) and Smoothened (Smo)* also result in HPE [4]. Collectively, this illustrates the importance of Hh signaling in embryonic craniofacial development and in the etiology of HPE. To date, mutational analyses of human HPE together with naturally occurring and engineered mouse mutants have identified the genetic lesions responsible for only about 20% of the cases of HPE. Hence it is critical to identify additional candidate genes for the majority of patients whose genetic lesions remain unknown.

Here we describe the *AP2-Cre* (“*Creface*”) mouse [15] as an insertion mutation in *Hhat*. *Hhat^Creface/Creface^* embryos exhibit holoprosencephaly; extensive apoptosis in the craniofacial mesenchyme; frontonasal, mediolateral, maxillary and mandibular prominence hypoplasia; patterning defects in the facial prominences and cranial ganglia; and diminished chondrogenesis and osteogenesis. Collectively these anomalies contribute to midfacial hypoplasia and agenesis of the jaw. In addition to holoprosencephaly and agnathia, *Hhat^Creface/Creface^* mutant embryos lack all but the ventral bones of the skull. We observed complete agenesis of the nasal, frontal, parietal, and interparietal bones amongst others, while the basioccipital, exoccipital, and basisphenoid bones were all severely hypoplastic. Thus we have identified *Hhat^Creface/Creface^* as a novel mouse model of acrania-holoprosencephaly-agnathia.

These phenotypes are consistent with perturbation of Hh signaling. Hh proteins are secreted glycoproteins that undergo autoproteolytic cleavage to generate their active forms. However dual cholesterol and palmitoyl post-translational lipid modification are also critical for proper Hh protein multimerization, distribution and activity [12]–[14]. *Hhat* encodes an acyltransferase and our results demonstrate that *Hhat* loss-of-function results in diminished palmitoylation of Shh. In support of our observations, *Skinny hedgehog* (*Skn*) which is also known as *Hhat*, was recently shown to catalyse the palmitoylation of all Hh proteins in invertebrates such as *Drosophila* [27], and mammals such as mice [25]–[29]. Furthermore, *Drosophila* mutants lacking Hh palmitoylation, exhibit patterning defects in Hh-responsive cells during wing and eye development, while limb development and spinal cord neurogenesis are disrupted in *Skn* mutant mice [25]–[29]. In agreement with this, *Hhat^Creface/Creface^* mutants exhibit a diminished gradient and range of Hh signaling as early as E9.5 as evidenced by the specific spatiotemporal loss of Hh production and downregulation of Ptch1. Neither Shh protein nor Ptch1-lacZ reporter activity could be detected in the ventral forebrain, floor plate or pharyngeal arch ectoderm of *Hhat^Creface/Creface^* embryos. During normal embryogenesis, *Shh* expression and subsequent Ptch1 activity in these tissues is induced by or dependent upon gradients of Shh emanating from other tissues such as the notochord and pharyngeal endoderm [30], [31],[38], [44]. Although some localized and possibly short-range Hh signaling remained intact, our data indicates that long-range Hh signaling gradients fail to be properly established during mammalian embryogenesis in the absence of Hhat. Thus the absence of palmitoylation prevents the formation of Hh multimeric complexes, subsequently limiting establishment of a Hh gradient. Therefore our analyses of *Hhat^Creface/Creface^* mouse embryos highlight the conserved requirement for Hhat in establishing Hh signaling gradients *in vivo* during mammalian embryogenesis and in particular during craniofacial development. Thus Hhat plays a critical role in the regulation of Hh signaling during embryogenesis.

### Patterning and cell survival are disrupted in *Hhat^Creface/Creface^* embryos

Hh signaling performs a number of key roles during craniofacial development. Hh is required to maintain the viability of neural crest cells [37] during their colonization of the facial prominences and also regulates patterning of the frontonasal region and pharyngeal arches [45], [46]. Interestingly, *Fgf8* is expressed in the proximal pharyngeal arch ectoderm [47], while *Bmp4* is expressed in the distal ectoderm and underlying mesenchyme [48]. *Fgf8* and *Bmp4* are critical regulators of jaw development as the loss of either gene results in specific jaw defects. Conditional deletion of *Fgf8* from the pharyngeal ectoderm results in the absence of molar teeth and most of the chondrocranial and dermatocranial elements that form the proximal jaw [49], [50]. In contrast, conditional inactivation of *Bmp4* in the mandibular ectoderm results in agensis of incisor teeth together with severe distal truncations of the jaw [42]. Thus, the jaw is patterned in a proximo-distal manner through Fgf and Bmp activity respectively [42], [50].

*Hhat^Creface/Creface^* embryos exhibit a complete absence of *Fgf8* and *Bmp4* expression in the pharyngeal arch ectoderm which demonstrates these genes are downstream targets of Hh signaling. Consistent with this idea, *Fgf8* and *Bmp4* expression is similarly diminished in *Shh* loss-of-function embryos [39], [51] and also as a consequence of pharyngeal endoderm ablation in chick embryos [44]. Taken together, this data indicates that Shh signaling from the pharyngeal endoderm is required for activation of Shh in the pharyngeal ectoderm and that this domain of activity in turn is essential for ectodermal *Fgf8* and *Bmp4* signaling during jaw development. Therefore, the presence of agnathia and anodontia observed in *Hhat^Creface/Creface^* embryos correlates with the loss of *Fgf8* and *Bmp4* activity in the pharyngeal arch ectoderm.

Fgf8 and Bmp4, both play critical roles in neural crest cell and craniofacial mesenchyme survival [42], [49] and examination of Erk1/2 and Smad1/5/8 activity as downstream readouts of Fgf and Bmp signaling respectively, indicated that Fgf signaling was primarily affected in *Hhat^Creface/Creface^* embryos. Interestingly, Shh is known to be required for the viability of neural crest cells [37] and activation and repression of Hh signaling *in vitro* increases and decreases Erk1/2 activity respectively [43]. Our analyses therefore suggest that Hh signaling acts through Fgf-Erk to promote neural crest cell viability and facial prominence outgrowth during craniofacial development. Consistent with this, Hhat is expressed at low but consistent levels throughout the facial prominences between E10.5–12.5 [52].

*Shh* loss-of-function embryos exhibit a reduction in the overall size of the brain, a single optic vesicle with agenesis of external eye structures, nasal proboscis and malformations of the craniofacial skeleton. *Shh* mutants also exhibit agenesis of all the calvarial bones irrespective of their neural crest or mesoderm origins [19]. The similarity in phenotypes between *Shh^−/−^* embryos and *Hhat^Creface/Creface^* mutants suggests that loss of Shh signaling in *Hhat^Creface^* mutants may also primarily account for the extensive defects in cranial vault. However, *Ihh* also plays an important role in craniofacial development during late gestation. Loss of Ihh results in reduced chondrocyte proliferation, an absence of bone calcification, hypoplasia of the cranial bones and diminished markers of osteodifferentiation during endochondral as well as membranous ossification [32]. [33]. Although there are three members of the *Hh* gene family, neither *Ihh* nor *Dhh* is appreciably expressed in craniofacial tissues prior to E12.5 [36], [53], [54]. Thus, whereas early neural crest cell survival and craniofacial morphogenesis depends primarily upon Shh signaling, later craniofacial differentiation and maturation requires both Shh and Ihh. Therefore during early embryogenesis, perturbation of *Shh* signaling is likely to be primarily responsible for apoptosis in the craniofacial mesenchyme and defects in facial prominence and pharyngeal arch patterning and outgrowth in *Hhat^Creface/Creface^* mutants. Consistent with this idea, perturbation of Hh signaling in neural crest cells through conditional deletion of *Smo* has no effect on neural crest migration but elicits a profound effect on the formation of skeletal and non-skeletal elements of the head [36].

Thus the combination of defects in patterning of the facial prominences, extensive apoptosis in the neural crest cell derived craniofacial mesenchyme and altered skeletogenic differentiation, underpins the craniofacial malformations in *Hhat^Creface/Creface^* embryos. In summary, we have identified *Hhat^Creface/Creface^* mice as a novel model of acrania-holoprosencephal-agnathia. Hhat palmitoylates Hh proteins and is critical for establishing proper gradients of Hh signaling during embryogenesis. Our work therefore highlights the importance of long-range Hh signaling in craniofacial development and suggests *HHAT* is a potential candidate in the etiology and pathogenesis of HPE and its associated congenital human disorders.

## Materials and Methods

### Mouse lines and maintenance

Mice were housed in the Laboratory Animal Services Facility at the Stowers Institute for Medical Research according to IACUC animal welfare guidelines. *Hhat^+/Creface^* were maintained on a CD1 background; genotyping of the *Hhat^+/Creface^* mice was performed as described below. The *Patched1(Ptch1)-LacZ* [20], *Rosa 26 Reporter (R26R)* [55], *Shh* [19], and *Wnt1-Cre* [56], [57] mice were maintained as previously reported. For embryo collection, the day of plug was noted as embryonic (E) day 0 and control embryos described in the analyses were either wild-type or heterozygous littermates. A minimum of 4 embryos were used for each assay performed unless otherwise stated. Embryo morphology was visualized by staining whole embryos with DAPI to label all cell nuclei and imaging fluorescent signal. Embryos were fixed in 4% paraformaldehyde overnight at 4°C then stained with 2 ug/ml DAPI in PBS and imaged by confocal microscopy. Scale bars designate magnification of embryos however in some cases may be a close approximation.

### Identification of the *Creface* transgene insertion site

The insertion location of the *Creface* transgene was identified using the Vectorette kit (Sigma, St. Louis, MO). A *Creface*;Vectorette DNA library was created using the EcoRI-Vectorette unit according to manufacturer's instructions. To amplify clone DNA containing the transgene and surrounding genomic region, the primer (5′-ACA TCT GGG GTG AAG GGA ATT AGG GAG TTG-3′) was used in addition to the Vectorette-specific primer to amplify DNA bands containing the integration site. A step-down PCR was used according to manufacturer's instructions; a 5 kb band, clone E1N3, was gel-purified (Gel Extraction Kit, Qiagen, Valencia, CA) and sequenced using the Vectorette sequencing primer. The blasted sequence aligned to intron 9–10 of the *Hhat* gene, indicating *Hhat* as the gene disrupted in *Creface* mutants.

Verification of transgene insertion site was performed using PCR in wild-type, heterozygous and mutant genomic DNA from embryos at E10.5. Amplification of the region spanning the insertion site used the transgene-specific primer (5′-TGG TTA CCT TCC TCC AGA TAG TATG-3′) and *Hhat*-specific primer (5′-CAC TTG CTA ACT AGA AGG AAC TTCC-3′) produced a 250 bp band; wild-type primers (5′-CCT GGG AAG GAA AAA CCA ATA TGTA-3′) and (5′-GGT CCT ATC ATG CTA CCA AGA AA-3′) amplified a 1.4 kb band. Samples were denatured at 94°C for 30 sec, annealed at 57°C for 30 sec, and extended at 72°C for 30 sec for 30 cycles for both reactions; PCR bands for the wildtype and mutant reactions were gel purified and sequenced, confirming *Hhat* as the gene disrupted by the *Creface* transgene.

To confirm the absence of *Hhat* mRNA in *Hhat^Creface/Creface^* mutants, RNA was isolated from wild-type and mutant embryos (RNeasy kit, Qiagen, Valencia, CA) and cDNA libraries were created using the Superscript first strand kit (Invitrogen, Carlsbad, CA). Primers (5′-AGG TTC TGG TGG GAC CCT GTGT-3′) and (5′-AGA AAG CAG TGT CCC CAA CAGG-3′) were used to amplify the full length *Hhat* mRNA in wild-type, heterozygous and mutant embryos. Primers to *glyceraldehyde 3-phosphate dehydrogenase (Gapdh)* (5′-AGC CTC GTC CCG TAG ACA AAAT-3′) and (5′-ACC AGG AAA TGA GCT TGA CAAA-3′) were used as an internal positive control.

### *In situ* hybridization

Embryos were collected as described above, fixed overnight in 4% paraformaldehyde (PFA) at 4°C, dehydrated in methanol (MeOH), and stored at −20°C until used in the staining protocol. *In situ* hybridizations were performed following the standard protocol described by [58]. Anti-sense digoxigenin-labeled mRNA riboprobes were synthesized for *Bmp4* (R. Arkell), *Crabp1* (S. Schneider-Maunoury), *Eya2* (P. Trainor), *Fgf8* (I. Mason), *Shh* (A. McMahon), *Six2* (P. Trainor), *Snail* (A. Nieto), *Sox9* (R. Krumlauf), and *Sox10* (M. Gassmann). *Hhat* forward 5′-CTG CGT GAG CAC CAT GTT CA-3′ and *Hhat* reverse 5′-TCT CCA CAG TGA CTC CCA GC-3′ primers were used to generate an exon 8–10 specific probe and whole embryos which was stained with *Hhat* antisense probe were mounted in Tissue Tek OCT (VWR, West Chester, PA) and sectioned at 20 µm thick to observe spatiotemporal activity.

### β-galactosidase staining

*Hhat^+/Creface^* mice were mated to *Rosa 26-lacZ reporter (R26R)* mice and embryos were collected at E9.5–11.5 as described above. Embryos were stained using the β-galacatosidase staining solution kit (Chemicon/Millipore, Billerica, MA) according to manufacturer's instructions. Briefly, embryos were fixed on wet ice for 20–30 min in the Tissue Fixative Solution, washed in Tissue Rinse Solution A for 30 min and Tissue Rinse Solution B for 5 min at RT. Embryos were incubated O/N at 37°C in Tissue Stain Solution with X-gal (40 mg/mL in DMF) (Invitrogen, Carlsbad, CA). After incubation, embryos were washed PBS and re-fixed overnight. Embryos were processed in paraffin, cut in 10micron sections, and counterstained in Nuclear Fast Red (NFR) (Sigma, St. Louis, MO).

For β-galactosidase staining on sections, E15.5 embryos were harvested and immersed in LacZ fixative (0.25%glutaraldehyde, 10% 0.5 M EGTA, and 10% 1 M MgCl_2_ in PBS) for 2.5 hrs at 4°C. Embryos were processed through a sucrose gradient (15% then 30% sucrose in PBS) and snap frozen in OCT. Eight micron sections were cut and re-fixed for 10 min at room temperature. Sections were then washed in LacZ wash buffer (0.2% MgCl_2_, 0.01% NaDOC, 0.02% NP40 in PBS) three times for 5 min. β-galactosidase staining was developed using a LacZ stain solution (0.002% Potassium ferrocyanide, 0.01% Potassium ferricyanide, 0.04% X-gal (25 mg/mL in DMF) in LacZ wash buffer) to the desired intensity. The sections were then rinsed three times in LacZ wash buffer for 5 min, counterstained with nuclear fast red and rinsed in distilled water for 1–2 min. Sections were mounted and photographed using a Ziess Axioplan microscope and processed using Photoshop CS2 (Adobe, San Jose, CA).

### Immunohistochemistry

For whole-mount staining using a neurofilament antibody, embryos were fixed in 4% PFA overnight at 4°C and dehydrated in a graded MeOH series. Dehydrated embryos were bleached in methanol∶DMSO∶30% H_2_O_2_ (4∶1∶1) for 5 hrs at room temperature and rehydrated in 50% methanol∶PBS and 15% methanol∶PBS, PBS for 30 minutes each. Embryos were blocked in PBSMT (2% milk powder, 0.1% Triton X-100 in PBS) twice for 1 hour at room temperature. Embryos were incubated in primary antibody to Neurofilament (2H3) diluted at 1∶250 O/N at 4°C in PBSMT. Embryos were rinsed in PBSMT and incubated in secondary antibody using a 1∶200 dilution in PBSMT of HRP-coupled goat anti-rabbit IgG (Jackson Immunoresearch, West Grove, PA). For color development, embryos were rinsed in PBSMT and PBT (0.2% BSA, Sigma, 0.1% Triton X-100) three times, 20 minutes each at room temperature and washed in 3,3-Diaminobenzidine (0.3 mg/mL) (Sigma, St. Louis, MO) in PBT for 20 minutes; 0.03% H_2_O_2_ was added and color was developed to the desired intensity.

For section immunohistochemistry, embryos were fixed overnight in 1% PFA at 4°C. Embryos were processed through a sucrose gradient (15%, then 30% sucrose in PBS), mounted in Tissue Tek O.C.T. (VWR, West Chester, PA) and sectioned at 10microns. Sections were rinsed three times in PBT (PBS with 0.1% Triton X-100) for 5 min and blocked in 10% goat serum (Invitrogen, Carlsbad, CA) in PBT for 1 hr at RT. Slides were incubated in primary antibody diluted in 10% goat serum/PBT overnight at 4°C. The mouse monoclonal antibodies to cleaved-Caspase-3 and pErk1/2 (Cell Signaling Technologies, Danvers, MA), phospho-Histone-3 (Upstate/Millipore, Billerica, MA) and Shh (5e1) were used at 1∶500 (Caspase-3, anti-pH3), 1∶250 (pErk1/2) and 1∶25 (5e1) respectively.

Slides were rinsed three times in PBT for 10 min each at room temperature and incubated in a goat anti-mouse Alexa 488 secondary antibody at 1∶250 (Molecular probes/Invitrogen, Carlsbad, CA) for 2 hours at 4°C. Sections were counterstained with a 1/1000 dilution of 2 mg/ml DAPI (Sigma, St. Louis, MO) in PBS for 5 minutes, followed by multiple rinses in PBS. Slides were then mounted with fluorescent mounting medium (DakoCytomation, Carpinteria, CA). All images were collected using a Ziess Axioplan microscope and processed using Photoshop CS2 (Adobe, San Jose, CA). Antibodies to Shh (5e1) and Neurofilament (2H3) were obtained from the Developmental Studies Hybridoma Bank developed under the auspices of the NICHD and maintained by the University of Iowa, Department of Biological Sciences (Iowa City, IA 52242).

### Cartilage and bone staining

Embryos were collected at E15.5 and 17.5 and fixed in 95% ethanol (EtOH) overnight. Embryos were washed in a stain solution containing 0.5% Alizarin red (Sigma, St. Louis, MO) and 0.4% Alcian blue 8× (Sigma, St. Louis, MO) in 60% EtOH overnight at room temperature. For E15.5dpc embryos, soft tissue was dissolved in 2% KOH for three hours and transferred to 0.25% KOH for 30 min. For E17.5 staining, the embryos were anesthetized in PBS for 1 hr at 4°C, fixed overnight in 95% EtOH, skinned and eviscerated prior to staining; all remaining soft tissue was dissolved in 2% KOH for six hours and transferred to 0.25% KOH for 30 min. Embryos were cleared in glycerol∶KOH (20%∶0.25%; 33%∶0.25%; 50%∶0.25%). Embryos were stored in 50% glycerol∶0.25% KOH until photographed.

### Palmitoylation assay

Immortalized mouse embryonic fibroblast (MEFs) derived from control and *Hhat^Creface/Creface^* mouse embryos were transfected *Shh* expression vector (gift from Dr. Pao-Tien Chuang) using Amaxa Nucleofactor (Amaxa, Germany) according to manufactures protocol. Twenty-four hours after transfection, cells were incubated with 50 µM Click-It palmitic acid (Invitrogen, Carlsbad,CA). After 12 hrs incubation, cells were washed three times with cold PBS and whole cell lysate was harvested using RIPA lyses buffer. Protein extracts were subjected to Click labeling reaction using Click-It protein reaction buffer kit (Invitrogen, Carlsbad,CA) in order to attach biotin to incorporated palmitic acid. Precipitated samples were again dissolved into RIPA buffer and mixed with mouse anti-*Shh* antibody (5e1) overnight at 4°C with rocking, and subsequently immunoprecipitated with protein G beads (Thermo scientific, Rockford, IL) with rocking for 2 hrs at room temperature. The beads were resuspended in SDS sample buffer and separated by SDS-PAGE and transferred onto a PVDF membrane (GE healthcare life sciences, Buckinghamshire, UK). The membrane was incubated with alkaline phosphatase streptavidin (Vector laboratories, Burlingame, CA) and treated with fluorescent substrate for alkaline phosphatase (Vector laboratories, Burlingame, CA), exposed to X-ray film to detect the palmitoylated Shh. Same membrane was incubated with anti Shh antibody (H-160; Santa Cruz Biotechnology Inc, Santa Cruz, CA) and subsequently HRP conjugated secondary antibody (Sigma, St.Louis, MO) in order to confirm the amount of Shh protein in each samples. The signals were detected with ECL plus western blotting detection system (GE healthcare life sciences, Buckinghamshire, UK) and exposed to X-ray film.

## Supporting Information

Figure S1 The *Creface* transgene disrupts *Hhat*. (A) Schematic representation of the structure of the *Creface* transgene [15] and its insertion at nucleotide position 22949 within intron 9 of *Hhat*. (B) Gel electrophoresis images demonstrating: (left) the specific amplification of an endogenous 1.4 kb fragment of intron 9 overlapping nucleotide position 22949 from control and *Creface^+/T^* genomic DNA but not from *Creface^T/T^* genomic DNA and (right) amplification of a 250 pb fragment encompassing part of the inserted transgene from *Creface^+/T^* genomic DNA and *Creface^T/T^* genomic DNA but not from control. (C) Gel electrophoresis images of RT-PCR amplification of a near full length 1.3 kb *Hhat* transcript and *Gapdh* control from E10.5 control and *Creface^+/T^* embryo mRNA and the absence of the *Hhat* transcript from *Creface^T/T^* embryo mRNA as a result of *Creface* transgene insertion in *Hhat.*
(JPG)

Figure S2 Patterns of neural crest cell migration. *In situ* hybridization for general markers of neural crest cells such as *Snail1* (A–D) and *Crabp1* (E–H) or lineage specific markers such as *Sox9* (I–L) and *Sox10* (M–P) in E8.75–9.0 control (A, B, E, F, I, J,M, N) and *Hhat^Creface/Creface^* (C, D, G, H, K, L, O, P) embryos reveals essentially normal patterns of neural crest cell formation, migration and lineage specification during early embryogenesis.
(JPG)

Figure S3 Indelible lineage tracing of neural crest cells using *Wnt1-Cre.* LacZ staining of *Wnt1-Cre;R26R* indelibly labeled migratory neural crest cells in E9.5 and 10.5 control (A, B) and *Hhat^Creface/Creface^* (C, D) embryos reveals essentially comparable patterns of neural crest cell migration (arrows) and colonization of the facial prominences. However, the facial prominences in *Hhat^Creface/Creface^* embryos are clearly hypoplastic (A–D), which is suggestive of a deficit in the number of neural crest cells. Furthermore mutant embryos exhibit aberrant fusion of the hypoglossal (IX, arrowhead) and vagus (X, arrowhead) nerves (B, D). Abbreviations: FNP, frontonasal process; r, rhombomere; V trigeminal ganglia; VII facial ganglia.
(JPG)

Figure S4 *Hhat^Creface/Creface^* embryos exhibit cranial ganglia defects. 2H3 anti-neurofilament immunostaining of E10.5 control (A) and *Hhat^Creface/Creface^* (D) embryos revealed (arrows and arrowheads) agenesis of the oculomotor nerve (III), hypoplasia of the trigeminal (V) and aberrant fusion of the hypoglossal (IX) and vagal (X) in mutant embryos. *In situ* hybridization for *Sox10* (B, E), and *Eya2* (C, F) respectively labeled neural crest and placodal progenitor cells highlighting their contributions to hypoplasia of the trigeminal and fusion of the hypoglossal (IX) and vagal (X) in mutant embryos.
(JPG)

Figure S5 Erk1/2 signaling is perturbed in *Hhat^Creface/Creface^* embryos. Immunostaining for phosphorylated Erk1/2 (brown) on transverse sections of E10.5 control (A) and *Hhat^Creface/Creface^* (B) embryos reveals that the domain and levels of pErk1/2 signaling are reduced in mutant embryos.
(JPG)
