# Supplementary figures and images for: Mutations in Hedgehog Acyltransferase (Hhat) Perturb Hedgehog Signaling, Resulting in Severe Acrania-Holoprosencephaly-Agnathia Craniofacial Defects
Source: PLoS Genet. 2012 Oct 4;8(10):e1002927. doi: 10.1371/journal.pgen.1002927 (PMC3464201; doi:10.1371/journal.pgen.1002927)

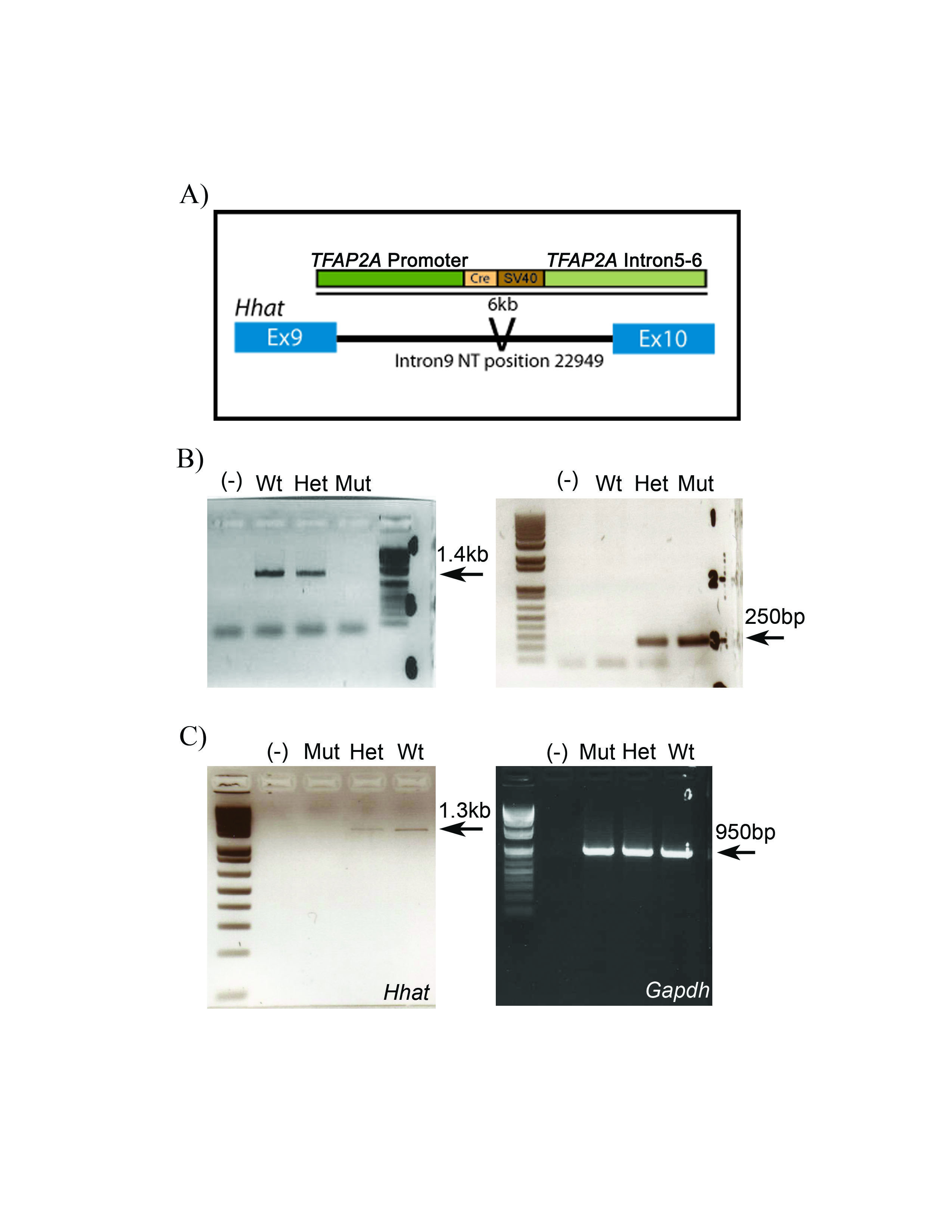

Supplement: Figure S1 — The Creface transgene disrupts Hhat. (A) Schematic representation of the structure of the Creface transgene [15] and its insertion at nucleotide position 22949 within intron 9 of Hhat. (B) Gel electrophoresis images demonstrating: (left) the specific amplification of an endogenous 1.4 kb fragment of intron 9 overlapping nucleotide position 22949 from control and Creface+/T genomic DNA but not from CrefaceT/T genomic DNA and (right) amplification of a 250 pb fragment encompassing part of the inserted transgene from Creface+/T genomic DNA and CrefaceT/T genomic DNA but not from control. (C) Gel electrophoresis images of RT-PCR amplification of a near full length 1.3 kb Hhat transcript and Gapdh control from E10.5 control and Creface+/T embryo mRNA and the absence of the Hhat transcript from CrefaceT/T embryo mRNA as a result of Creface transgene insertion in Hhat. (JPG) [file pgen.1002927.s001.jpg]

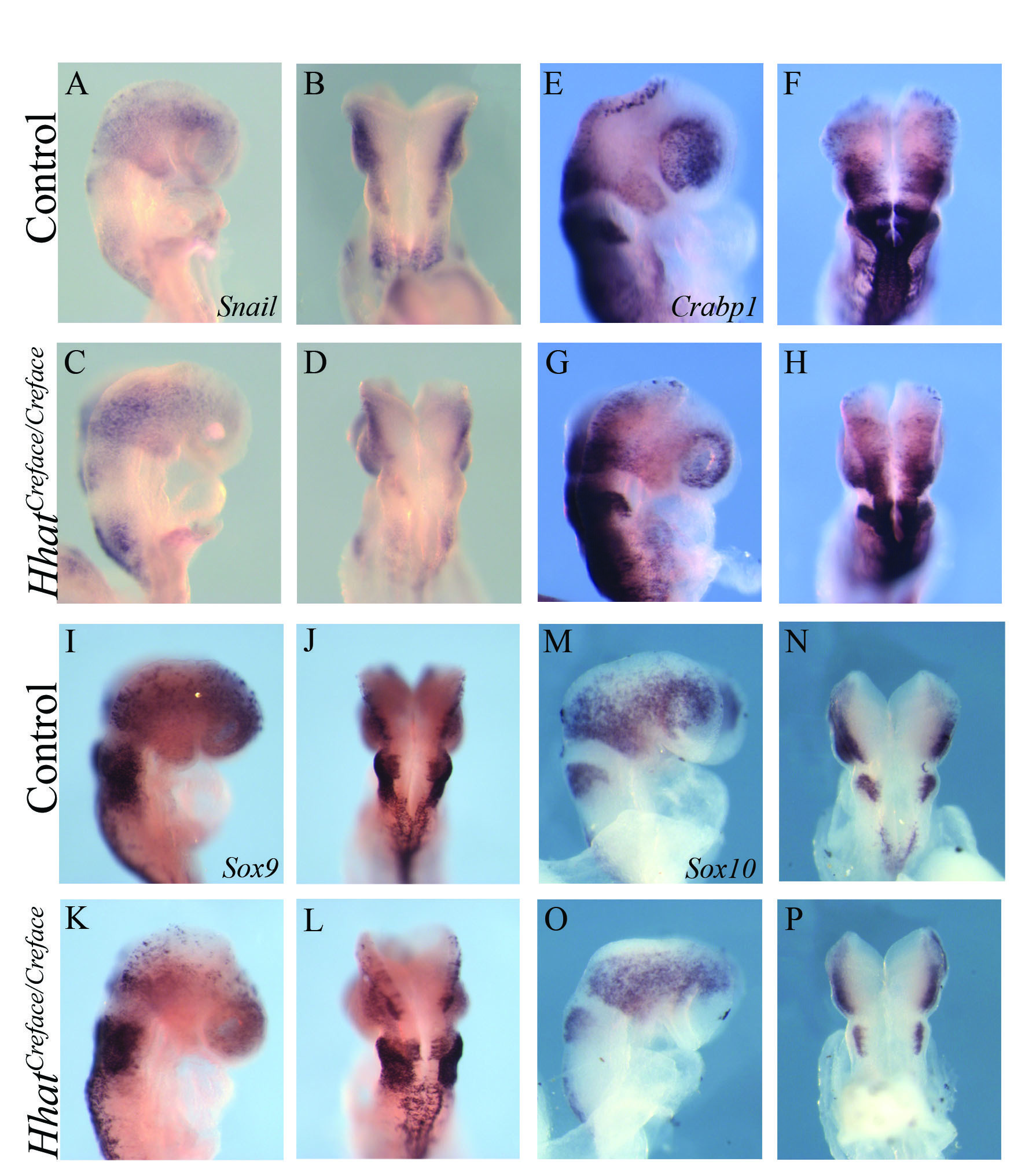

Supplement: Figure S2 — Patterns of neural crest cell migration. In situ hybridization for general markers of neural crest cells such as Snail1 (A–D) and Crabp1 (E–H) or lineage specific markers such as Sox9 (I–L) and Sox10 (M–P) in E8.75–9.0 control (A, B, E, F, I, J,M, N) and HhatCreface/Creface (C, D, G, H, K, L, O, P) embryos reveals essentially normal patterns of neural crest cell formation, migration and lineage specification during early embryogenesis. (JPG) [file pgen.1002927.s002.jpg]

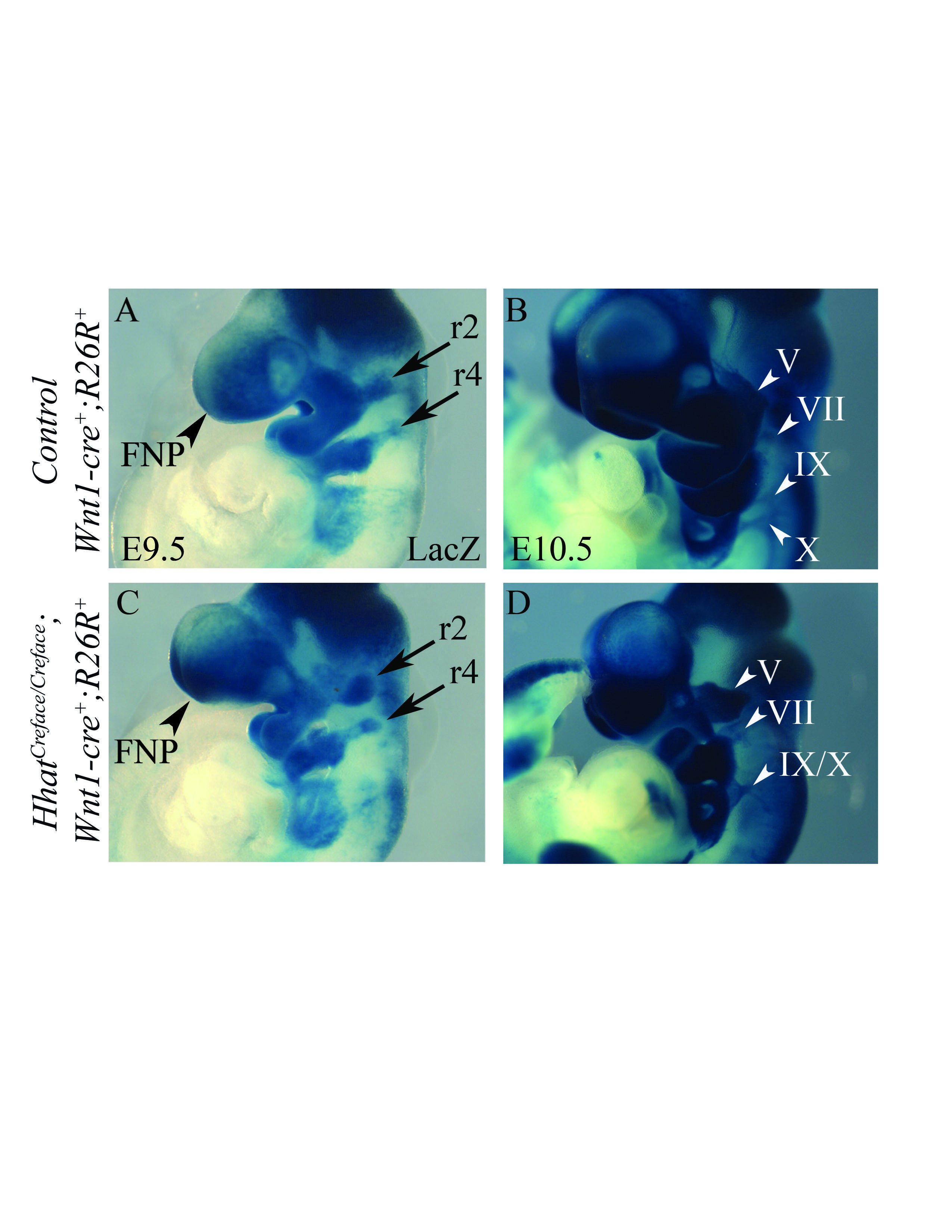

Supplement: Figure S3 — Indelible lineage tracing of neural crest cells using Wnt1-Cre. LacZ staining of Wnt1-Cre;R26R indelibly labeled migratory neural crest cells in E9.5 and 10.5 control (A, B) and HhatCreface/Creface (C, D) embryos reveals essentially comparable patterns of neural crest cell migration (arrows) and colonization of the facial prominences. However, the facial prominences in HhatCreface/Creface embryos are clearly hypoplastic (A–D), which is suggestive of a deficit in the number of neural crest cells. Furthermore mutant embryos exhibit aberrant fusion of the hypoglossal (IX, arrowhead) and vagus (X, arrowhead) nerves (B, D). Abbreviations: FNP, frontonasal process; r, rhombomere; V trigeminal ganglia; VII facial ganglia. (JPG) [file pgen.1002927.s003.jpg]

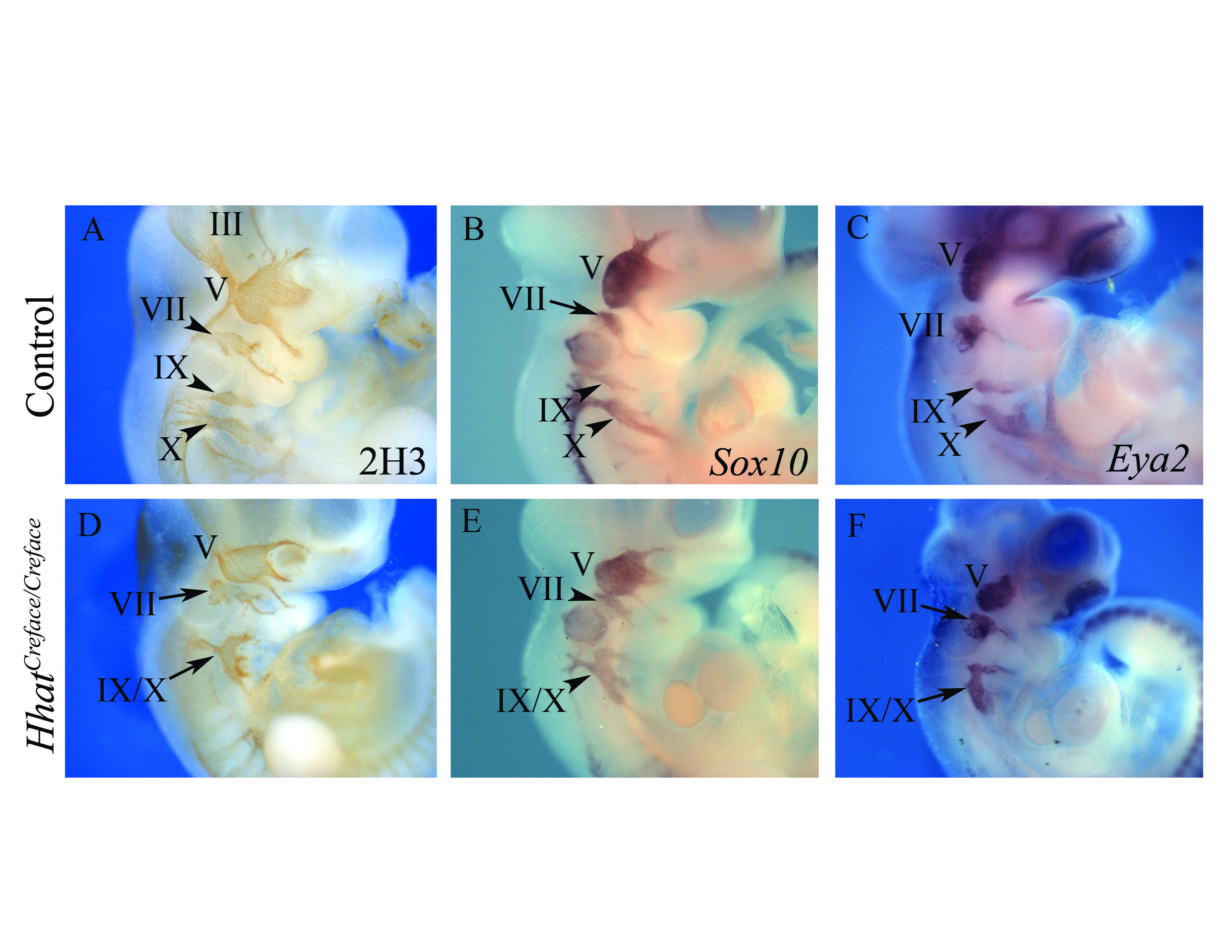

Supplement: Figure S4 — HhatCreface/Creface embryos exhibit cranial ganglia defects. 2H3 anti-neurofilament immunostaining of E10.5 control (A) and HhatCreface/Creface (D) embryos revealed (arrows and arrowheads) agenesis of the oculomotor nerve (III), hypoplasia of the trigeminal (V) and aberrant fusion of the hypoglossal (IX) and vagal (X) in mutant embryos. In situ hybridization for Sox10 (B, E), and Eya2 (C, F) respectively labeled neural crest and placodal progenitor cells highlighting their contributions to hypoplasia of the trigeminal and fusion of the hypoglossal (IX) and vagal (X) in mutant embryos. (JPG) [file pgen.1002927.s004.jpg]

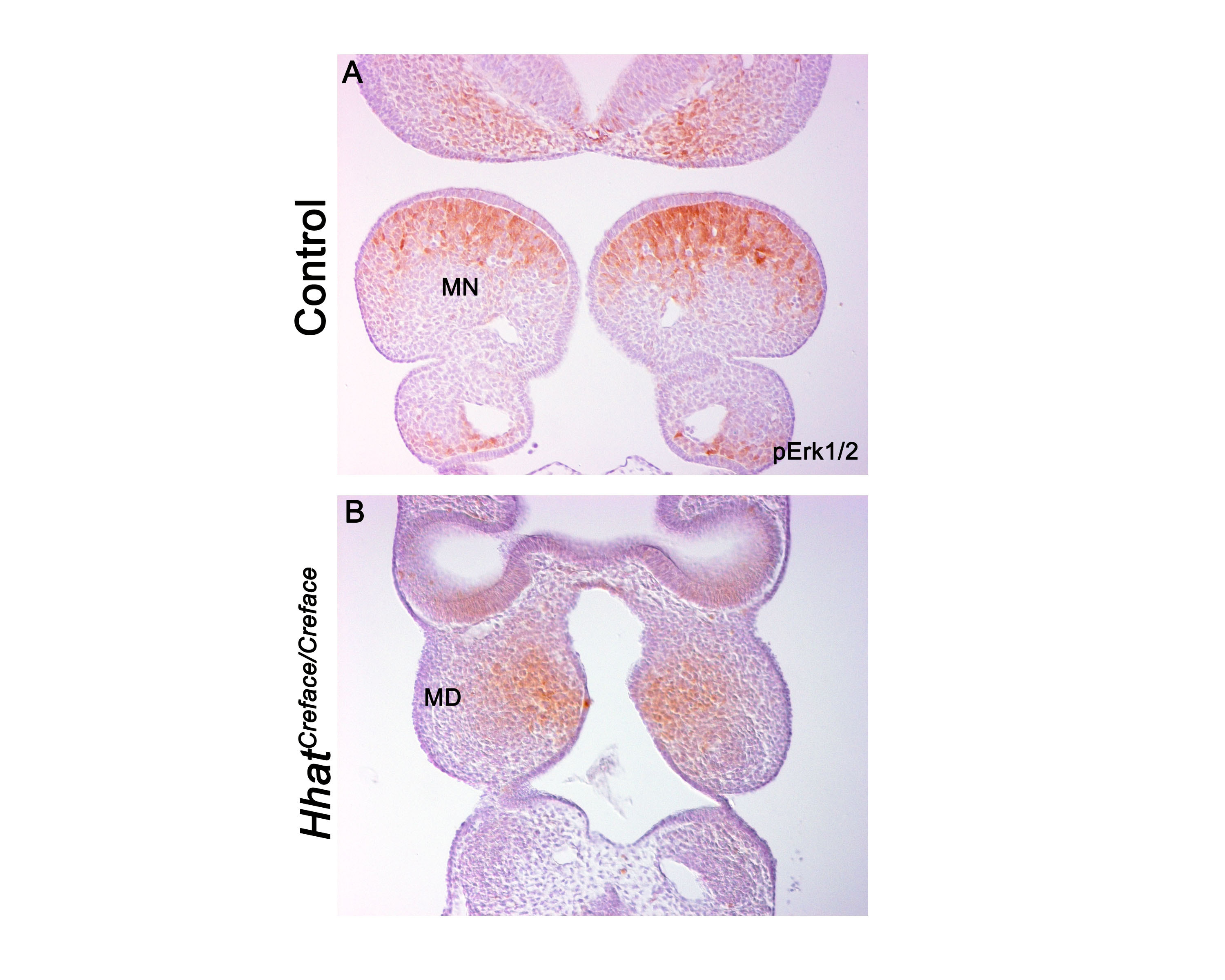

Supplement: Figure S5 — Erk1/2 signaling is perturbed in HhatCreface/Creface embryos. Immunostaining for phosphorylated Erk1/2 (brown) on transverse sections of E10.5 control (A) and HhatCreface/Creface (B) embryos reveals that the domain and levels of pErk1/2 signaling are reduced in mutant embryos. (JPG) [file pgen.1002927.s005.jpg]
